# Factors influencing persistence of *Legionella pneumophila* serogroup 1 in laboratory cocultures

**DOI:** 10.1186/s12866-014-0249-8

**Published:** 2014-10-03

**Authors:** Angelo G Solimini, Alessia Cottarelli, Lucia Marinelli, Maria De Giusti

**Affiliations:** Department of Public Health and Infectious Diseases, Sapienza University of Rome, Piazza A. Moro 5, 00185 Rome, Italy

**Keywords:** *Legionella pneumophila*, *Pseudomonas aeruginosa*, Biofilm, Planktonic phase, Heterotrophic plate count, Drinking water, Hospital, Iron

## Abstract

**Background:**

Risk for infections from *Legionella pneumophila* for immunocompromised individuals increases greatly when this species is present within the biofilm of the water distribution systems of hospitals or other health facilities. Multiplication and persistence of *Legionella* may dependent also upon planktonic growth in alternative to sessile growth. Here we compared the persistence of *L. pneumophila* serogroup 1 in experimental planktonic co-cultures subsided with iron, *Pseudomonas aeruginosa* and other non Legionella bacteria (quantified as Heterotrophic Plate Count, HPC at 37°C), isolated from drinking water sources of a large hospital.

**Results:**

Concentrations of *L. pneumophila* showed a decreasing pattern with incubation time in all co-cultures, the degree of reduction depending on the experimental treatment. In co-cultures with added *P. aeruginosa*, no *L. pneumophila* was detectable already after 4 days of incubation. In contrast in co-cultures without *P. aeruginosa*, HPC but not iron were significant factors in explaining the pattern of *L. pneumophila*, although the HPC effect was different according to the incubation time (HPC x time interaction, p < 0.01).

**Conclusions:**

Our results highlight the need of controlling for both HPC and metal constituents of the water systems of buildings used by individuals at particular risk to the effects of *Legionella* exposure.

## Background

*Legionella pneumophila* is an ubiquitous microorganism present in both natural and artificial water systems that may cause a potential life threatening form of pneumonia called Legionnaires’ disease. Especially when colonizing the water distribution systems of buildings of hospitals or other health facilities, the risk for infections for immunocompromised individuals increases greatly. In such artificial aquatic systems, the biofilm covering the interior of pipelines and, more in general, the various plumbing elements represent relatively nutrient-rich spots where *Legionella* (and other bacteria) can attach and multiply [1,2]. Biofilm is a matrix composed by heterogeneous aggregates of bacteria, fungi, protozoa, and algae embedded into extracellular polymeric substances [3]. As a result, many engineered water systems such as air conditioning cooling towers, water boilers, whirlpools and spas, drinking water distribution networks, shower heads, and dental-unit water lines provide an environment conducive to the growth and multiplication of *Legionella* species ([2] and references therein).

The survival of microorganisms in man-made systems results from the (interactive) effects of several factors which depend themselves from the structure and management of the water network. Those factors include temperature [4], pipe material [5], nutrient levels [6-8], frequencies, type and concentration of disinfectants [9,10], water velocity [11] and hydraulic conditions [12]. Among the micronutrients, lower levels of certain metals enhance growth of *L. pneumophila* [13] and especially iron has been linked to *L. pneumophila* extracellular growth, intracellular replication, and virulence [14,15]. As iron availability in water distribution systems may be linked with the older age and metal constituents of pipes [13], corrosion products are important factors in the survival and growth of *L. pneumophila* in artificial habitats [13,16].

Several empirical environmental studies have examined the simultaneous effects of chemical elements, biofilm biomass (also quantified as heterotrophic plate counts, HPC, at 22°C and/or 37°C) and other factors on *L. pneumophila* presence [17-19] with inconsistent results [20,21]. For example, occurrence of *Legionella* spp in samples from public and private structures (including hospitals, hotels and private houses) was positively associated with HPC at 22°C (but not with HPC 37°C), manganese (but not iron) and negatively with copper and higher water temperatures [22].

In experimental cocultures the growth and/or persistence of *L. pneumophila* was positively associated with several isolates of heterotrophic bacteria from potable water [23-26] as well as negatively with others, that were able to inhibit *Legionella* growth [27-29]. In particular, *P. aeruginosa*, another species often present in artificial water systems, seems able to secrete inhibitory substances like lactones [30] and resulted not conductive to *L. pneumophila* [26].

More generally, *Legionella* persistence in artificial systems was linked to its capacity of parasitize several species of protozoans and to multiply within them [2], to efficiently exploit available nutrients from the surroundings [2], and to obtain nutrients with necrotrophic feeding [31]. It has been suggested that multiplication of *Legionella* may dependent upon planktonic growth in alternative to sessile growth (e.g. within biofilm), because the planktonic phase would increase the likelihood of protozoan encounters [32]. If *Legionella* can persist in a planktonic phase also in absence of protozoans remains unclear, and this information is relevant for adopting the necessary management strategies in water systems where the presence of *L. pneumophila* increases the infection risk.

The aim of this study is to test the persistence of *L. pneumophila* in experimental planktonic cocultures subsided with iron, *P. aeruginosa* and other non Legionella bacteria (quantified as HPC at 37°C) isolated from the drinking water of a large hospital. In this system, despite the risk management interventions with regular disinfections, *L. pneumophila* has been isolated several times at several water point of use in the past few years [33].

## Methods

### Study design

To test the effect of subsidy of iron, other non Legionella bacteria (measured as HPC at 37°C) and *P. aeruginosa* co-presence on *L. pneumophila*, we set up a full factorial design with iron (2 levels: 0 and 0.1 mg/l initial concentrations), HPC at 37°C (3 levels: 0, 10 and 100 CFU/ml initial concentrations) and *P. aeruginosa* (2 levels: 0 and 10 CFU/ml initial concentrations) as factors. All factor level combinations were prepared in duplicate samples. A total of 24 tubes were generated (see below preparation of co-cultures) inoculating a fixed concentration of *L. pneumophila* in each test tube and measuring its density over four time points (at 0, 4, 8, 16 days from inoculum) as described below.

### Bacterial strains

The *Legionella* strain used in this study was a control strain of *L. pneumphila* serogroup 1 (American Type Culture Collection,ATCC, 33152, Oxoid Ltd., UK). This strain was used because the same serogroup was isolated from the tap water of the hospital where the other bacteria were also obtained. This strain was grown in Buffered Charcoal Yeast Extract (BCYE) agar supplemented with L-cysteine (SR110C Oxoid), Ferric pyrophosphate 0,25 g/L and selective antibiotics (Polymyxin B, Anisomycin and Vancomycin; SR0118E Oxoid) incubated at 36 ± 1°C with 2.5% CO_2_ for 10 days.

The bacteria used in co-cultures were isolated from tap water of the hospital according to the standard method (UNI EN ISO 6222:2001). The following species were isolated, identified (Vitek 2 Compact bioMerieux) and maintained in monospecific colonies on a Standard Plate Count agar (Oxoid): *Brevundimonas diminuta/vescicularis*, *Sphingomonas paucimobilis*, *Acinetobacter baumanni*, *Pseudomonas alcaligenes. P. aeruginosa* was isolated from the tap water of a different point of use of the hospital according to the standard UNI EN 12780:2002 and maintained on Pseudomonas agar base (CM0559, Oxoid) with selective supplement (SR0103E, Oxoid).

### Preparation of co-cultures

A single colony of *L. pneumophila* was transferred in 100 ml of buffered yeast extract broth (yeast extract: 10 g/L; glicine: 3 g/L) supplemented with L-cysteine (SR110C) and incubated overnight at 36 ± 1°C with constant shaking [30].

Before the inoculum, the optical density (OD) was measured at 600 nm (OD600; Coulter Du-530, Beckman) to determine the concentration of bacteria in 1 ml of suspension. The concentration of *Legionella* in the suspension was then adjusted till an OD600 of 0.19 by adding fresh buffered yeast extract broth.

A single colony of *P. aeruginosa* was transferred into 30 ml of sterile physiological saline solution and incubated overnight at 36 ± 1°C with constant shaking. The other biofilm components (HPC) were similarly incubated by transferring a single colony from each monospecific culture in 30 ml of steril physiological saline solution. The concentrations of *P. aeruginosa* and of HPC were determined similarly to *L. pneumophila* by measuring OD600 and adjusting the concentration in the suspensions using predetermined relationships between OD600 and viable counts.

Co-cultures were set up according to the study design by adding 1 ml of *L. pneumophila* suspension, 0.1 ml of HPC and 0.1 ml of *P. aeruginosa* and iron (0.1 mg/L, added as ferrous sulfate eptaidrate (Cpachem), to 9 ml of sterile deionized water with resistivity 18,2 MΩ × cm and conductivity 0,055 μS/cm a 25°C and left in the dark at room temperature (approximately 23°C).

### Cells enumeration

All the microbial parameters in each different co-culture were monitored at different times in accordance to the study design (at 0, 4, 8, 16 days from inoculum), to determine the variation of concentration by the viable count method on selective agar.

Concentration of *L. pneumophila* was determined by seeding serial dilutions of 0.1 ml from each co-culture on the selective agar BCYE supplemented with L-cysteine. The plates in duplicate were incubated at 36 ± 1°C with 2.5% CO_2_ for 10 days and the reading was performed at intervals of 2–3 days. The detection limit of this method was 10 CFU/ml.

HPC concentration was determined by the inclusion method by seeding serial dilutions of 1 ml of the microcosm solution on Stadard Plate Count Agar and incubated at 36 ± 2°C for 48 hours. The concentration of *P. aeruginosa* was determined by seeding serial dilutions of 0.1 ml of the microcosm on Pseudomonas agar base with supplement and incubated 36 ± 2°C for 48 hours.

### Statistical analysis

Differences in HPC and *P. aeruginosa* (Log_10_ transformed) concentrations between subsidised co-cultures controls at each time from inoculum were assessed with an Anova and subsequent multiple comparisons with Bonferroni correction. Pattern of *Legionella* concentration (Log_10_ transformed) between different treatments over the 4 times was modelled using a linear model for correlated data [34]. Time from inoculum, iron, HPC were entered as fixed effects and all second and third order interactions tested. A heterogeneous variance-covariance structure was used to account for non homogenous variances in (Log_10_ transformed) *L. pneumophila* concentration between times. Additionally, autoregressive error structure was used to handle correlated errors deriving from the repeated measures design [34]. The model with heterogeneous variance covariance structure and autoregressive errors was preferred over simpler models (with homogeneous variance and/or no autoregressive errors) after model comparisons using AIC and likelihood test [34]. All analysis was carried out using R 3.0.2 and packages nlme 3.1 [35] and lsmeans 2.05 [36].

## Results

*P. aeruginosa* and HPC concentrations (Table 1) were different between treatments and controls at time 0, according to the experimental design (Anova, all comparisons at time 0, p < 0.05). After 4 days of incubation, the mean concentrations of HPC and *P. aeruginosa* in co-cultures with *L. pneumophila* reached concentrations >1 × 10^6^ CFU/ml and increased reaching concentrations >1 × 10^8^ CFU/ml at 16 days of incubation, being similar in all experimental treatments (Anova, all comparisons at times 4, 8, 16, p > 0.05).

**Table 1:** **Average concentrations (Log**_**10**_ **transformed) and standard error (SE) of heterotrophic plate count (HPC) and** ***P. aeruginosa*** **at different times from inoculum in cocultures with** ***L. pneumophila*** **srg 1 with different treatment**

**Co-culture initial condition**					
**P. aeruginosa (CFU/ml)**	**Fe (mg/l)**	**HPC (CFU/ml)**	**Time from inoculum (day)**	**HPC Log**_**10**_ **(CFU/ml) (SE)**	***P.aeruginosa*** **Log**_**10**_ **(CFU/ml) (SE)**
0	0	0	0	0	0
			4	0	0
			8	0	0
			16	0	0
0	0.1	0	0	0	0
			4	0	0
			8	0	0
			16	0	0
0	0	10	0	1.6 (0.2)	0
			4	6.0 (0.0)	0
			8	8.0 (0.1)	0
			16	7.5 (0.1)	0
0	0.1	10	0	2.0 (0.2)	0
			4	6.0 (0.0)	0
			8	8.1 (0.1)	0
			16	8.3 (0.1)	0
0	0	100	0	3.0 (0.0)	0
			4	6.0 (0.0)	0
			8	8.9 (0.0)	0
			16	8.5 (0.0)	0
0	0.1	100	0	3.0 (0.0)	0
			4	6.0 (0.0)	0
			8	8.3 (0.1)	0
			16	8.2 (0.1)	0
10	0	0	0	0	1.3 (0.0)
			4	0	8.3 (0.0)
			8	0	8.7 (0.0)
			16	0	8.2 (0.0)
10	0.1	0	0	0	1.7 (0.0)
			4	0	8.3 (0.0)
			8	0	8.5 (0.0)
			16	0	8.9 (0.0)
10	0	10	0	2.0 (0.2)	1.2 (0.0)
			4	6.0 (0.0)	8.3 (0.0)
			8	8.5 (0.0)	8.5 (0.0)
			16	8.3 (0.0)	8.3 (0.0)
10	0	100	0	3.2 (0.0)	1.7 (0.0)
			4	6.0 (0.0)	8.3 (0.0)
			8	8.5 (0.0)	8.5 (0.0)
			16	8.7 (0.0)	8.2 (0.0)
10	0.1	10	0	2.1 (0.1)	1.4 (0.1)
			4	6.0 (0.0)	8.3 (0.0)
			8	8.4 (0.0)	8.6 (0.1)
			16	9.8 (0.0)	9.3 (0.0)
10	0.1	100	0	3.2 (0.0)	1.8 (0.0)
			4	6.0 (0.0)	8.3 (0.0)
			8	8.4 (0.0)	8.6 (0.0)
			16	9.8 (0.0)	9.5 (0.0)

**Figure 1:**
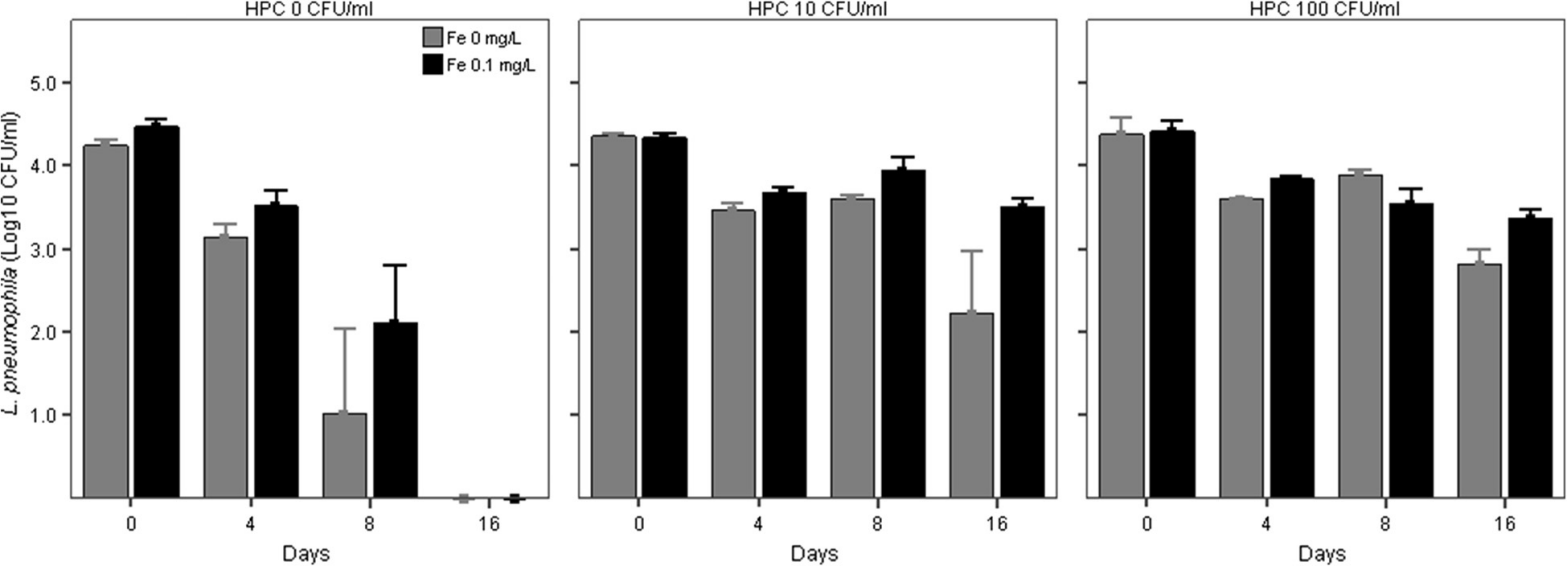
**Persistence of** ***L. pneumophila*** **srg 1 in co-cultures with different initial heterotrophic plate counts at 37°C (HPC) and iron (Fe) subsidy in absence of** ***P. aeruginosa*** **.** Bars show mean and 1 standard error.

**Figure 2:**
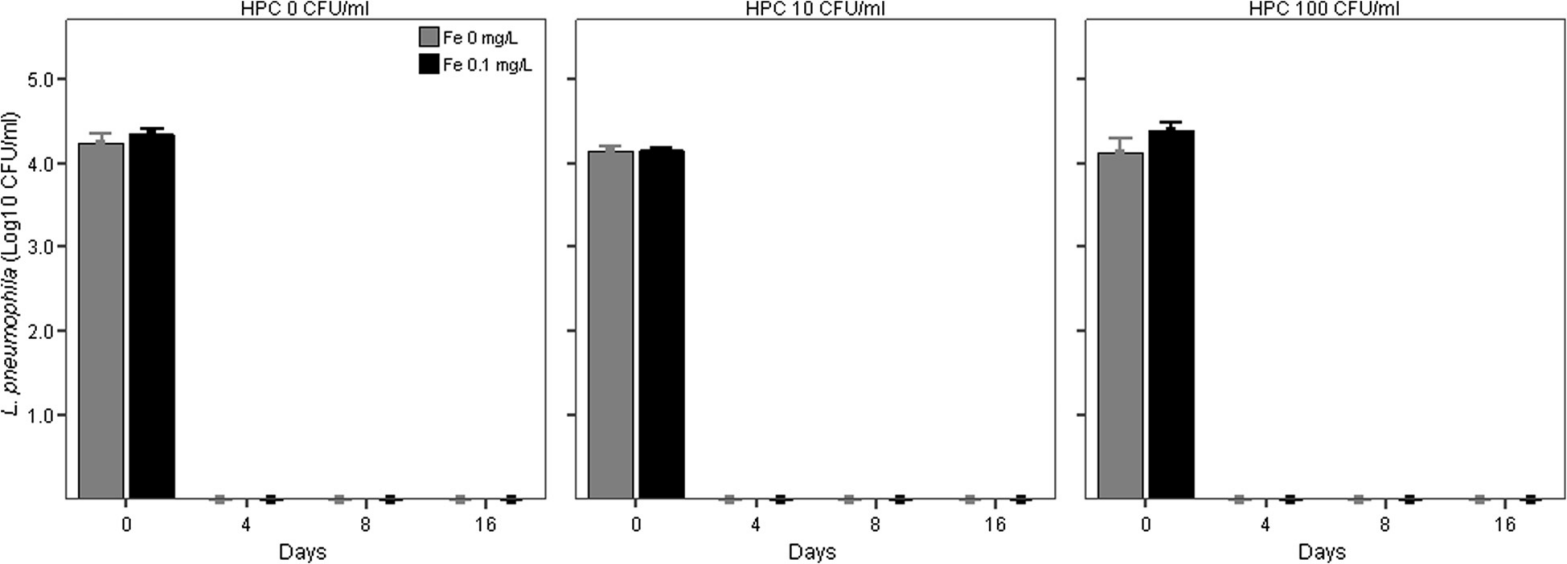
**Persistence of** ***L. pneumophila*** **srg 1 in co-cultures with different initial heterotrophic plate counts at 37°C (HPC) and iron (Fe) in presence of** ***P. aeruginosa*** **.** Bars show mean and 1 standard error.

Concentrations of *L. pneumophila* showed a decreasing pattern with incubation time in all co-cultures (Figure 1 and 2), the degree of reduction in concentrations depending on the experimental treatment. In the co-cultures with added *P. aeruginosa*, at time 0 *L. pneumophila* concentrations were not different between co-cultures supplemented with different initial iron (p = 0.10) and HPC concentrations (p = 0.15) but no *L. pneumophila* was detectable already after 4 days of incubation (Figure 2). In contrast, in co-cultures without *P. aeruginosa* (Figure 1), HPC but not iron treatments were significant factors in explaining the pattern of *L. pneumophila* (Table 2). The HPC effect was different according to the incubation time (HPC × time factor, p < 0.01, Table 2). After 4 days of incubation, *L. pneumophila* concentrations were higher than controls only in co-cultures supplemented with HPC at initial concentration of 100 CFU/ml (Table 3, mean difference = −0.39 CFU/ml, p < 0.05) while starting from day 8 *L. pneumophila* concentration was higher than controls in all HPC level treatments (Table 3, all differences p < 0.05).

**Table 2:** **Effect of experimental treatments on (Log**_**10**_ **transformed)** ***L. pneumophila*** **srg 1 in cocultures without** ***P. aeruginosa***

**Term**	**Numerator degrees of freedom**	**F statistics**	**P-value**
Intercept	1	1136.11	<0.01
HPC	2	0.36	0.70
Fe	1	2.21	0.14
Time	3	39.07	<0.01
Fe × HPC	2	0.86	0.43
HPC × time	6	5.39	<0.01
Fe x time	3	0.72	0.54
Fe × HPC × time	6	1.15	0.34

Time: time from inoculum (4 levels: 0, 4, 8, 16 days), Fe: iron (2 levels: 0 and 0.1 mg/L initial concentrations), HPC: heterotrophic plate count (3 levels: 0, 10 and 100 CFU/ml initial concentrations).

**Table 3:** **Multiple pairwise comparisons of** ***L. pneumophila*** **srg 1 least squares means between co-cultures with different initial levels of heterotrophic plate count (HPC) and controls (no HPC added) at different times from inoculum**

**Time from inoculum (day)**	**Treatment comparison (HPC initial concentrations; CFU/ml)**	**Mean difference of marginal means control - treatment (standard error)**	**t-ratio**	**P-value**
0	0– 10	0.01 (0.10)	0.08	0.99
	0– 100	−0.4 (0.11)	−0.37	0.93
4	0– 10	−0.24 (0.12)	−2.00	0.12
	0– 100	−0.39 (0.14)	−2.84	0.02
8	0– 10	−2.21 (0.39)	−5.68	<0.001
	0– 100	−2.15 (0.45)	−4.79	<0.001
16	0– 10	−2.86 (0.34)	−8.52	<0.001
	0– 100	−3.09 (0.39)	−7.96	<0.001

## Discussion

Our results show a positive association between *L. pneumophila* concentration and the supplementation of HPC and iron and a negative association with *P. aeruginosa* presence. The main reason of this positive association is probably the fact that bacteria of the genus *Legionella* depends on other microbes to survive in nutrient poor environments such as the experimental planktonic co-cultures. In water distribution systems and other man-made aquatic systems, *Legionella* interacts with other microrganisms by adopting several survival strategies [17,37]. The primary survival strategy is linked to *Legionella* ability of deriving the necessary nutrients (carbon, nitrogen, and amino acids) from dead microrganisms (via necrophilic exploitation, [31]) or by efficient uptake of nutrients excreted by other bacteria [2]. Second, *Legionella* is also able to parasitize a wide range of protozoans and to multiply within them [2,38]. The same capacity is shown when *Legionella* infects alveolar macrophages of humans, causing pulmonary infections. Third*, Legionella* seems also able to enter a planktonic growth phase (opposed to the benthonic attached phase), in response to low nutrient conditions within the biofilm [2]. Additionally, biofilm embedded *Legionella* cells may have enhanced protection from chlorine based biocides that are commonly used to manage the infection risks in private and public buildings [1,18,39].

In our experiment, *Legionella* was able to persist in planktonic cultures supplemented with HPC but not in those supplemented with iron alone or with *P. aeruginosa* (regardless co-presence of iron and/or HPC). Therefore, although iron has been linked to *L. pneumophila* extracellular growth, intracellular replication, and virulence [14,15], it was unable to sustain *L. pneumophila* without the contemporary presence of other microrganisms, which were probably the source of other essential nutrients. This result is also coherent with the observation that supplemented iron increases *Legionella* persistence in HPC supplemented co-cultures beyond the levels reached in co-cultures with HPC only (Table 3). It has been previously shown that iron (but also other elements necessary for *Legionella* growth like other metals or nutrients like phosphorus) in bioavailable form can be released by certain pipe constituents via biofilm mediated corrosion or from deposits of materials in stagnant portions of the water distribution system [40,41]. Additionally, certain chemical compounds released from pipes can interfere with the chlorine based disinfectant decreasing its effects on biofilm cells [42,43]. Therefore, when managing *Legionella* risk, the effect of pipe material and the physical structure of the water distribution network should be taken into account.

In our co-cultures the presence of *P. aeruginosa*, regardless of the level of supplements HPC and iron inhibited *L. pneumophila* growth from the early beginning of the experiments. This inhibitory effect on *Legionella* growth has been shown for several microrganisms residing in the same biofilm matrix. For example, [29] suggested that up to 32% of HPC bacteria (including *Aeromonas*, *Vibrio*, *Pseudomonas* and *Pseudomonas*-like strains) isolated from chlorinated drinking water were able of inhibiting or slowing the growth of *Legionella* species. Another study showed that *L. pneumophila* cannot attach in biofilms with pre-existent *P. aeruginosa* [26]. Although many mechanisms involved in the processes of microbial interference are only partially known [17], the production of inhibitory bacteriocins was linked to *Legionella* growth suppression in several earlier studies. For example, [28] showed that a bacteriocin producer like *P. fluorescens*, can inhibit both the formation and the stability of *L. pneumophila* biofilms. Similarly, it has been shown that *Legionella* can attach to *P. aeruginosa* monospecies biofilms but cannot persist for more than 2 days of incubation [26], possibly because of the effect of homoserine lactones produced by *P. aeruginosa* [30]. It remains to be clarified if *Legionella* can persist in viable but not cultivable form (VBNC, a form still able to cause human infections, [44]) even in presence *P. aeruginosa*. This may explain the reason why we found positive samples to *Legionella* VBNC in a previous cross sectional survey of at point of use water sources in the same hospital from where the biofilm of this experiment was obtained.

## Conclusion

Many studies examined so far the relative effect of several factors that are reported empirically to increase the likelihood of *Legionella* presence in water distribution systems in settings like hospitals or other health care facilities where a formal risk assessment and management is a priority [1]. The results of our experiments support those studies reporting a positive association with HPC counts and iron subsidy and a strong negative association with *P. aeruginosa*, also in static flow conditions. Notably, all the microbial colonies used in this study were previously isolated from the hospital drinking water at point of use, and our data provide specific insights for the management of *Legionella* risk in this hospital setting. Although a confirmatory study should be designed in microcosms with dynamic flow conditions, our results highlight the need of controlling for both HPC and metal constituents into water systems of buildings at particular risk for the effects of *Legionella* exposure on humans such as hospitals and care homes for the elderly. Future studies should also elucidate the effect of protozoans in modifying the persistence of *L. pneumophila* in co-cultures as well as in water distribution systems. Moreover, additional effort should also investigate what environmental factors of the drinking water systems trigger the entrance to and the exit from VBNC state (including escape strategy from repeated chlorination cycles [39]). This understanding could lead to improved control measures for *L. pneumophila* in hospital settings.
